# The usefulness of lactate dehydrogenase measurements in current oncological practice

**DOI:** 10.1186/s11658-020-00228-7

**Published:** 2020-06-09

**Authors:** Agata Forkasiewicz, Maja Dorociak, Kamilla Stach, Piotr Szelachowski, Renata Tabola, Katarzyna Augoff

**Affiliations:** 1 Department of Surgical Education, Wroclaw Medical University, ul. Sklodowskiej-Curie 66, 50-369 Wroclaw, Poland; 2 Department of Biochemistry, Wroclaw Medical University, Wroclaw, Poland; 3 Second Department and Clinic of General and Oncological Surgery, Wroclaw Medical University, Wroclaw, Poland

**Keywords:** Lactate dehydrogenase, LDH isoforms, Warburg effect, Tumor markers

## Abstract

One of the hallmarks of cancer cells is increased energy requirements associated with the higher rate of cellular proliferative activity. Metabolic changes in rapidly dividing cancer cells are closely associated with increased uptake of glucose and abnormal activity of lactate dehydrogenase (LDH), which regulates the processing of glucose to lactic acid. As serum LDH levels were found to be commonly increased in cancer patients and correlated with poor clinical outcome and resistance to therapy, the determination of LDH has become a standard supportive tool in diagnosing cancers or monitoring the effects of cancer treatment.

The aim of this review is to summarize the current knowledge about methods and the practical utility for measuring both the total LDH and LDH isoenzymatic activities in the diagnosis, prognosis and prediction of cancer diseases.

**This article was specially invited by the editors and represents work by leading researchers**.

## Introduction

From a metabolic point of view, the most striking and common feature of cancer cells is the production, despite the availability of oxygen, of a large amount of lactic acid, resulting from the enhanced glycolysis, which is positively correlated with the upregulation of glucose transporter-1 (Glut1) and therefore an increase in glucose uptake [1–5]. Although Warburg was the first to observe this phenomenon in the 1920s, its molecular basis and relationship with cancer genetics are still incompletely understood. There are different transcription factors, including Myc genes/proto-oncogenes or the hypoxia inducible factors HIF-1 and HIF-2, that are known to regulate glycolysis through binding to a highly conserved carbohydrate response element (ChoRE) with the consensus sequence CACGTG or hypoxia-responsive elements (HREs) located in the promoters of genes encoding glycolytic enzymes such as *LDHA*. These factors are activated in response to changes in oxygen saturation and tension or glucose access and have been shown to be closely associated with cancer growth and progression [6–10]. It is known that glycolysis is necessary for the G1 to S phase transition in the cell cycle and downregulation of glycolysis stops the cell in the G1 phase of the cell cycle, indicating that glucose metabolism plays an important role in the regulation of cell proliferation [6, 9, 11]. It is also known that in the presence of oxygen glycolysis is inhibited in normal cells [12]. However, the reason for escaping the “Pasteur effect” in cancer cells and the preference for low-energy processes based on conversion of glucose into lactate, even in the presence of oxygen, remains unexplained.

## Lactate dehydrogenase

### Catalytic properties

Lactate dehydrogenase (LDH, L-lactate, NAD+ oxidoreductase, EC1.1.1.27) is a family of at least six NAD + -dependent isoenzymes (LD1-LD5 and LD6/LDX). It is recognized as being one of the most common enzymes in nature. It belongs to the class of oxidoreductases and it is characteristic for the final stage of anaerobic glycolysis. LDH catalyzes the reversible conversion of pyruvate to lactate with the concomitant oxidation/reduction of NADH to NAD^+^ [13]. Activation of LDH is an ordered sequential process. The binding of a substrate to LDH is preceded by the formation of the LDH/NADH binary complex in which His 195 and Asp 168 residues seem to play a key role. It is followed by conformational changes in which the mobile hinged loop, formed by a group of surface amino acid residues (98–110) with Arg 109 hydrogen bonded to the carbonyl of the substrate, closes over the substrate binding pocket, comprising residues 163–247 and 267–331, to allow for interactions between the cofactor and the substrate, facilitating on-enzyme catalysis [14–16].

### Metabolic significance

The unique role of lactate dehydrogenase is particularly evident under limited oxygen conditions, when oxidation of NADH in the respiratory chain is not possible. The reduction of pyruvate catalyzed by LDH allows the regeneration of NAD+ molecules, which are needed for the continuous generation of ATP to maintain glycolysis [17]. Therefore, under hypoxia, the reduction of pyruvate to lactate allows cells with high glycolytic activity to survive an anaerobic episode. However, since the use of lactate for further metabolic processes can only take place after it is converted back to pyruvate, lactate production is a dead end for the cells’ metabolism [2]. In addition, when pyruvate is not used as a substrate in the citric acid cycle and oxidative phosphorylation, the amount of energy released per mole of oxidized glucose is reduced. Thus, in anaerobic conditions, 18 times more glucose molecules are used to generate the same amount of energy as in aerobic conditions [2, 17].

**Fig. 1:**
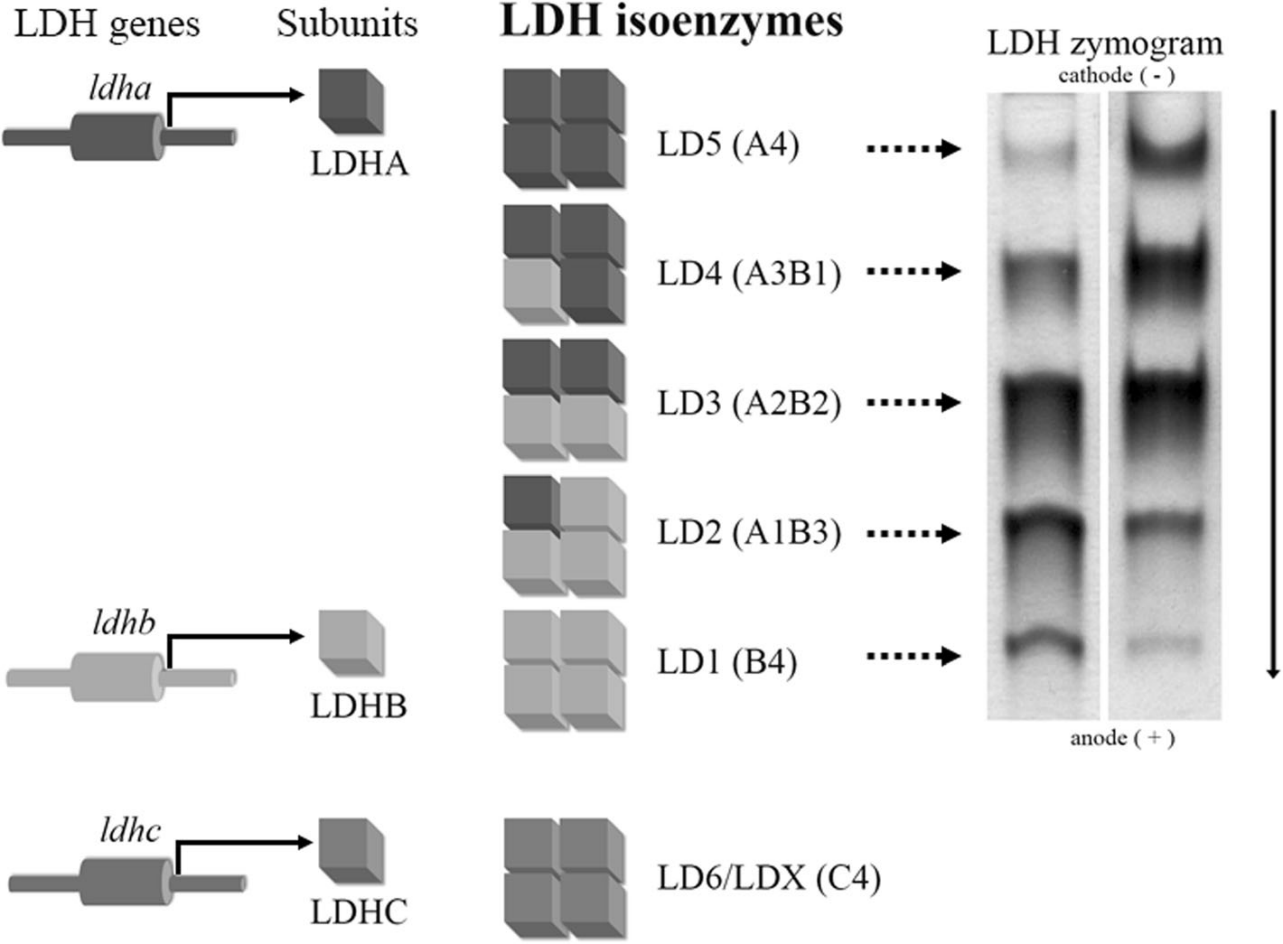
Scheme showing the LDH reaction. LDH catalyzes the reversible conversion of pyruvate to lactate with NADH as a cofactor during glycolysis*.* The LD5 isoform preferentially catalyzes the reduction of pyruvate into lactate and thus prevents entry of pyruvate to the tricarboxylic acid (TCA) cycle. The NADH oxidation allows the regeneration of NAD+ molecules, which are needed for the continuous generation of ATP to maintain glycolysis. By contrast, the LD1 is predisposed to convert lactate into pyruvate with the concomitant generation of NADH

Active LDH isoforms (LDHi) are homo- or heterotetrameric combinations of two subunits (Fig. 1) with the molecular weight of approximately 35,000 Da each: A (“M” – muscle) and B (“H” – heart), encoded by two separate genes, *ldha* and *ldhb*, located on chromosomes 11p15.4 and 12p12.2-p12.1, respectively [9, 18–23]. The combinations of A and B proteins into tetrameric complexes result in the five isoforms B4, A1B3, A2B2, A3B1 and A4, named LD1 to LD5 or LDH1 to LDH5, respectively [13]. A third type of LDH subunit, known as C, is encoded by the *ldhc* (or *ldhx*) gene located on chromosome 11p15.5-p15.3 and is relatively homologous structurally to the A (75.3% identity) and B subunits (69.8% identity) [14, 24]. The C subunit assembles only into homotetramers and was found to be sperm- and testis-specific [23, 25]. It is interesting that the widespread existence of alternative splicing of the *ldhc* gene was observed also in human cancers, with high frequency in lung cancer, melanoma, and breast cancer, but never in healthy control tissues [25, 26].

**Fig. 2:**
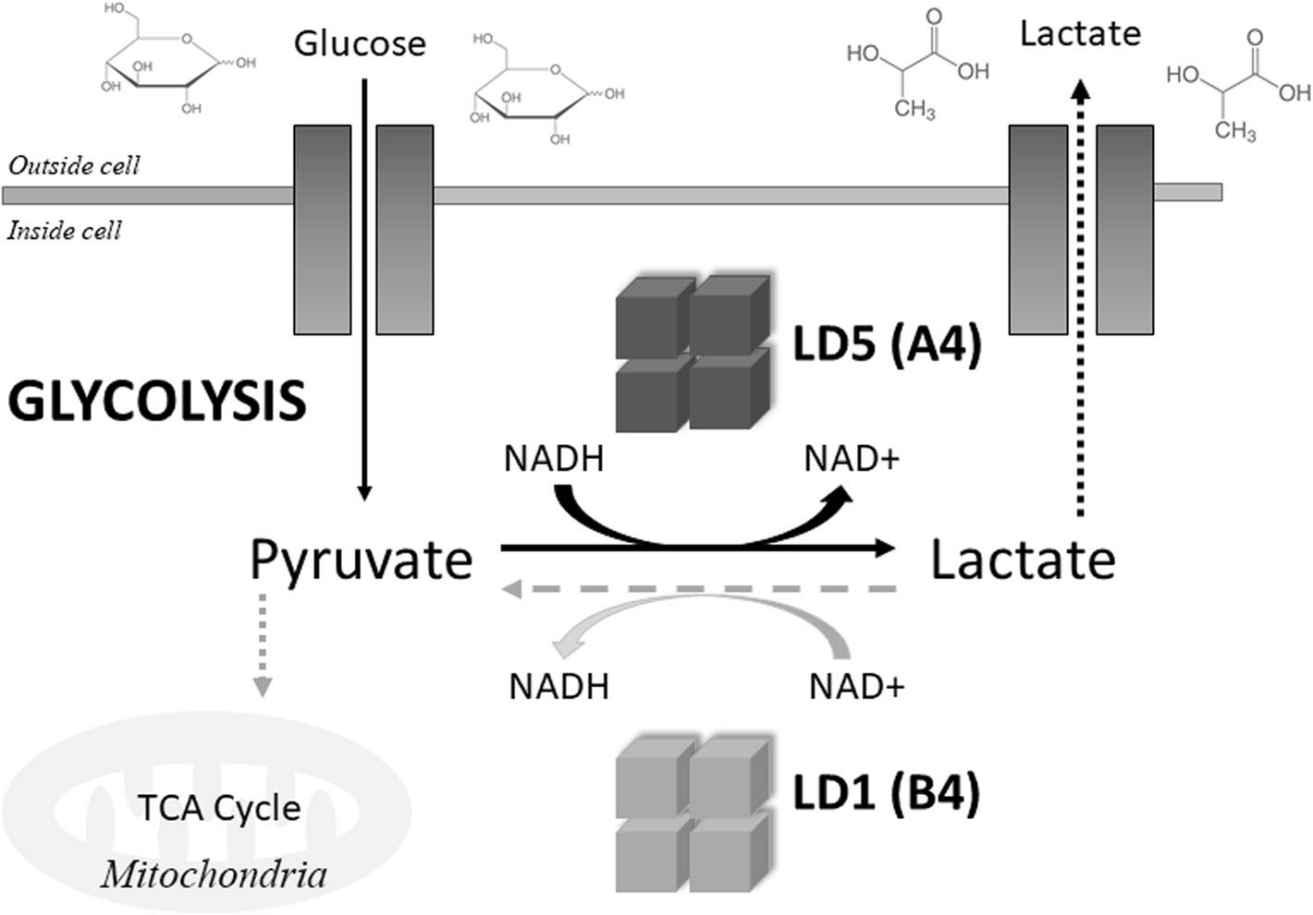
Scheme presenting LDH isoforms. The *ldha* and *ldhb* genes encode LDHA and LDHB polypeptide subunits. The combinations of these two subunits into active tetramers results in the five LDH isoforms B4, A1B3, A2B2, A3B1 and A4, named LD1 to LD5, respectively. A third type of LDH subunit, known as C, is encoded by the *ldhc* gene. The LDHC subunit assembles only into homotetramers to form the LD6 (or LDX) isoform. The multiplicity of LDH molecular forms can be revealed on the basis of their different mobility in gel electrophoresis. The LD1 isoform has the highest and LD5 the lowest migration rate towards the anode, as demonstrated in zymograms

Although all isoforms catalyze the same biochemical reaction, they vary in molecular structure, substrate affinity, temperature sensitivity and tissue specificity [19, 22, 27]. Each subunit determines the specific metabolic characteristics of isoforms. According to the “aerobic-anaerobic theory”, the contribution of individual isoforms to the total LDH activity is determined by the metabolic function of the individual tissue, with each tissue being able to adapt a specific enzymatic profile for its needs [19, 27]. Consequently, different amounts of LDHi were found in various tissues [18]. The A subunit predisposes LDH isoforms to convert pyruvate into lactate, while the isoforms in which the B subunit predominates kinetically favor the conversion of lactate to pyruvate (Fig. 2) [9, 19, 28]. Although there are exceptions to this rule (an example is the human liver – the organ, although remarkably aerobic, is characterized by the dominant activity of the A4 (LD5) form – the fact is that the expression of individual isoforms in cells reflects the metabolic state of tissues. This is consistent with the experimental finding that maturing tissues and tissues that undergo neoplastic transformation are characterized by significant changes in isoenzymograms [19, 27].

### Molecular properties

Differences between isoforms are a consequence of existing structural differences between LDH subunits and their quantitative ratio in active, tetrameric forms of the enzyme [27]. The variation in the amino acid composition of A and B polypeptides is the source of significant differences in the primary structure, mostly homotetrameric A4 (LD5) and B4 (LD1) isoenzymes [19]. As demonstrated, these differences appear much larger when comparing both isoforms derived from one organism than those that occur between isoforms of different species, even very taxonomically distant [19, 23, 27]. Using interspecific cell hybrids, it was shown that LDH subunits from different species were capable of associating to form functional enzymes. It indicates that despite interspecies differences in the amino acid composition of the subunits, the required structure of each of them, enabling the formation of an active tetramer, remains preserved [19].

### Differences between isoforms

Comparison of tertiary structure of the three homotetrameric isoforms (B4, A4 and C4, commonly named LD6 or LDH 6) has indicated many significant differences between these proteins, with the C4 (LD6) form being more different from A4 (LD5) and B4 (LD1) than these two isoforms between each other. It was shown that antibodies directed against the mouse C4 (LD6) isoform did not cross-react with mouse A4 (LD5) and B4 (LD1) but bound to C4 (LD6) forms derived from other species [29]. The C4 (LD6) isoform is characterized by substitutions in the amino acid sequence that result in conformational changes, which gives C4 (LD6) unique structural and physical properties. The C4 (LD6) isoenzyme displays a significantly higher affinity for lactate, thermostability as well as a broader range of substrate recognition than other LDH isoforms [30].

Differences in the stability of each specific subunit have a significant impact on the differences in temperature sensitivity of individual isoenzymes [19, 23]. The B subunit predisposes tetramers to maintaining conformation and catalytic integrity over a wide temperature range. Isoform B4 (LD1) maintains enzymatic activity at an elevated temperature to about 60 °C, under conditions in which the A4 (LD5) form is quickly inactivated. These differences also apply to hybrid forms according to the principle in which the natural logarithm of the rate constant for thermal inactivation of individual isoenzymes is inversely proportional to their electrophoretic mobility. The B4 form is also more stable at low temperatures (≤ 8 °C), compared to the A4 (LD5) form [18, 31]. Individual isoforms also show varying sensitivity to excess pyruvate, which is closely related to the heterogeneous affinity of these forms for the substrate. Each of the isoenzymes has its optimal activity at a different pyruvate concentration. Thus, the optimum concentration of pyruvate for A4 (LD5) is about five times higher than for B4 (LD1), while it is strongly directly proportional to the pH and temperature of the environment [18, 19]. It is known that at constant pH and temperature, the greater the electrophoretic mobility and the smaller the Michaelis constant (Km), the lower the concentration of pyruvate that is needed to inhibit the activity of a given form, and the lower the pyruvate concentration optimum [14, 18]. The situation is similar in the case of lactate. Form A4 (LD5) can function in an environment with a much higher concentration of lactate than form B4 (LD1), and the relative Km values ​​determined for both substrates clearly indicate that the A4 (LD5) enzyme binds both pyruvate and lactate with lower affinity than the B4 (LD1) isoform [14, 32–34]. The major difference in the catalytic activity is probably associated with a substitution of Ala by Gln residues (in the A and B subunits, respectively) located close to the binding site of the coenzyme phosphates [35]. Comparison of the amino acid composition of the two isoforms has shown that they differ in their surface charges. The overall net charge for the B4 isoform was calculated to be − 6, while the net charge of the A4 form was found to be + 1 (per subunit) [14].

The LDH isoforms also differ in their antigenic specificity. These differences are evidenced by the lack of positive cross-reaction of antibodies obtained for both LDH homotetramers, A4 (LD5) and B4 (LD1) [23, 27]. The use of antibodies against A4 (LD5) in the reaction with a mixture of LDH isoenzymes causes inhibition of both A4 (LD5) and A3B1 (LD4) isoforms with the same effect. These specific antibodies were also found to inhibit the activity of A2B2 (LD3) and A1B3 (LD2), but the inhibitory effect was significantly smaller and correlated with their mobility in the electric field [18]. The observed significant changes in the LDH isoenzymogram in cells exposed to changes in oxygen pressure in the environment seem to be closely related only to the expression of the A polypeptide. Under such conditions, the synthesis of the B subunit is virtually unchanged. According to Fonda and Kapan, even a slight decrease in oxygen pressure caused an increase in the total amount of lactate dehydrogenase, correlated with an increase in the contribution of the A subunit. Thus, the decrease in oxygen pressure causes the expression of LDH isoforms of this type, which function better in anaerobic conditions. A good example illustrating this principle is the renal parenchyma, in which along with a decrease in oxygen pressure towards its central part, an increase in the percentage of the A4 (LD5) isoform in total LDH activity is observed [18].

### Similarities between isoforms

The A4 and B4 homotetramers have similar values of the sedimentation coefficient (s), despite structural differences, which may indicate that these enzymes have the same surface area. There are also no significant differences in the domain structure, association of subunits, active-site regions, or the molecular mass of LDH isoforms, whose average value was determined to be 147,000 ± 7000 Da [14, 18].

## Determination methods

LDH is released from cells in response to cell damage, causing its baseline level to rise in the extracellular space and the bloodstream or other body fluids. Therefore, LDH has been recommended as a general marker of cell/tissue injury or to help identify the type of cells or tissues that are damaged [36]. Various testing methods are applied to measure LDH, both quantitatively and qualitatively, either as a total protein or as an individual LDH isoenzyme.

As the reduced form of nicotinamide adenine dinucleotide (NADH), which servs as a cofactor in LDH-related conversion of pyruvate to lactate, can absorb light at 340 nm and have intrinsic blue fluorescence, the detection of NADH absorption or fluorescence was found to be a useful tool to determine the activity of LDH. This method, however, is not sensitive enough to detect NADH at a low concentration. Recently some electrochemical methods that use graphene-based nanomaterials or modified graphite and carbon electrodes have been shown as very attractive for detection of NADH with high sensitivity [37, 38].

The most common laboratory tests to measure the level of lactate dehydrogenase (LDH) are based on colorimetric methods by monitoring absorbance in the range 430–550 nm, using various tetrazolium salts that are reduced to formazan, displaying a broad spectrum of colors from dark blue, deep red, to orange, by NADH with phenazine methosulfate serving as an intermediate electron carrier [39]. Other tests use specific fluorescent probes to generate fluorescent or bioluminescent products [40, 41]. All these tests can be used to quantify LDH activity in a wide variety of samples including serum, tissue extracts, cell line lysates or cerebrospinal, peritoneal and pleural fluids [42–46]. They are also commonly used for monitoring the cytotoxicity and cell viability in cell cultures [47].

The fractionation of LDH isoforms can be performed by native electrophoresis on different media such as agarose gel, cellulose-acetate or polyacrylamide gel, ion exchange chromatography or affinity chromatography [18, 19, 22–24, 27, 31, 48]. Since the B subunit is more negatively charged than the A subunit, the LD1 isoenzyme, composed of four B subunits, migrates faster towards the anode than the LD2, LD3, LD4 and LD5 isoforms. The LD5 fraction, known as the cathodic fraction, composed of four A subunits, is the slowest LDH isoform [20, 22, 23, 27, 31]. In extreme cases, next to the LD5 form, a very characteristic, additional band showing lactate dehydrogenase activity, has also been observed. This “extra” band has been identified as a marker of poor prognosis of liver cell damage [23, 32].

The use of guanidine isothiocyanate (GITC) to inhibit activity of LD2-LD5 isoforms was found to be a useful tool to test the activity of the B4 homotetrameric form by measuring the formation rate of NADH at 340 nm wavelength, using spectrophotometry [49].

Detection and quantification of LDH isoenzymes may be performed using assays based on immunological techniques such as Western blot, immunohistochemistry (IHC) or the enzyme-linked immunosorbent assay (ELISA) [50, 51]. The expression of LDH subunits can be additionally measured at the mRNA level by real-time PCR. LDHA mRNA was found to be highly expressed in invasive cancers and positively correlated with tumor growth [5].

Additionally, it was demonstrated that extracellular lactate, the end product of LDH activity, can be used for the assessment of human T cell proliferation and activation [52].

## Clinical significance of lactate dehydrogenase in oncology

### LDH as a diagnostic and prognostic marker for cancers

The widespread presence of LDH in cells, isoenzymatic tissue specificity and changes in the expression of individual subunits of this enzyme, associated with changes in tissue metabolic functions (e.g. in pathological conditions), have made LDH a significant diagnostic parameter in myocardial infarction, liver diseases with hepatic cell damage such as acute liver failure (ALF), hemolytic anemia and various types of myopathies [18, 23, 28, 53–57]. With the appearance of Warburg’s reports of increased glycolysis activity in transformed cells, the attention of researchers focused on the prognostic value of serum LDH levels in cancer patients (Table 1). However, more recent studies have shown that LDH is a non-specific diagnostic marker for cancers [23, 54, 58]. LDH activity determined in urine, although proposed as a marker of bladder or kidney neoplastic changes, was found to be elevated also in cases of upper urinary tract infections [85]. Similar difficulties arise when attempting to differentiate benign and malignant tumors based on total LDH activity [54]. However, in cases of blood or breast cancers, it is suggested to determine total LDH activity as a prognostic factor [9, 61]. The value of total lactate dehydrogenase activity also seems to be a useful marker in making decisions about therapeutic management of testicular cancer patients [64, 65]. Low LDH activity is associated with a high probability of complete remission of the disease and a good prognosis [9, 23, 54].

**Table 1:** The summary of some of published studies presenting data on LDH as a potential diagnostic, prognostic and predictive marker for cancers

CANCER TYPE	DIAGNOSTIC MARKER	PROGNOSTIC MARKER	PREDICTIVE MARKER
*Urinary tract cancers*: bladder cancer, Wilms’ tumor	↑ total LDH activity and ↑ LD5 level in urine [23, 54]	↑ LDHi levels in urine [58]	↑ total LDH activity in serum [58, 59]
*Breast cancers*: triple-negative breast cancer (TNBC)	↑ cathodic LDHi [60]	↑ total LDH activity in serum [61]	↑ total LDH activity in serum [62, 63]
*Germ cell cancers*: testicular cancer, ovarian cancer	↑ LD1 level and ↑ cathodic LDHi levels in serum [21, 23, 58]		↑ total LDH activity in serum [58, 64, 65]
*Gastrointestinal tract cancers*: colorectal cancer (CRC), gastric cancer (GC), pancreatic cancers (PC), esophageal squamous cell carcinoma (ESCC), Hepatocellular carcinoma (HCC)	↑ LD5 level in serum and ↑ LDHA/LDHB ratio [58, 66]	↑ total LDH activity in serum and ↑ cathodic LDHi [66–70]	↑ cathodic LDHi levels and ↑ total LDH activity in serum [58, 69–71]
*Lung cancers*: small-cell lung cancer (SCLC), epidermal growth factor receptor mutation-positive non-small cell lung cancer, Non-small cell lung cancer (NSCLC)	↑ LD5 level in cancer tissue and ↑ cathodic LDHi in serum, and pleural fluid [20, 58, 72]	↑ total LDH activity in serum and ↑ plasma LDH activity [58, 73, 74],	↑ total LDH activity in serum [73, 75–78]
Prostate cancer (PC)		↑ total LDH activity in serum [79]	↑ total LDH activity in serum [58]
Brain cancer	↑ total LDH activity in cerebral cyst fluids [80]		
*Oral cancers*: oral squamous cell carcinoma (OSCC)		↑ total LDH activity in serum [81]	↑ LDB expression in tumor tissue [82]
*Blood cancers*: Hodgkin’s lymphoma (HL), non- Hodgkin’s lymphoma (NHL), Burkitt’s lymphoma, Chronic granulocytic leukemia (CGL)	↑ total LDH activity and ↑ LD3 level in serum [54]	↑ total LDH activity in serum [23, 54, 58]	
Ewing’s sarcoma		LDHA expression in tumor tissue [23, 83]	
Malignant teratoma	↑ LD1 level in serum [84]		

In the case of Wilms’ tumor, LDH is used as a marker in both diagnosis and monitoring of the response to therapy [23, 54, 59]. Increased total LDH activity is observed in most tumor tissues or even precancerous lesions, although this is not the rule [81]. Total LDH activity does not change significantly, e.g. in lung or stomach cancers [20, 67]. It was suggested that the increase in LDH activity is influenced by the presence of necrotic processes within the tumor tissue [21]. On the other hand, it is known that cancerous tissues, in comparison with normal tissues, are characterized by the presence of a much larger number of connective tissue components, which, according to some authors, may be the cause of false low results [67].

### LDH isoenzymes in cancers

Most human cancer tissues, in addition to increased total LDH activity [18, 20], show changes in the ratio of lactate dehydrogenase isotypes, which tend to increase the expression of forms with the predominance of the A subunit [22, 60]. Significant changes in favor of LDH-A isoenzymes have been observed in many malignancies, particularly those located in the thyroid, colon, uterus and ovaries, stomach, kidneys, central nervous system and lungs [9, 19–22, 54, 66–68, 72, 80, 84]. Less frequently, significant changes in the number of LDH-B isoforms are observed. In the case of benign lesions, lactate production is much lower than in malignant tumors, and the percentage of individual isoforms does not differ significantly from that determined in normal tissues from which the lesions originate [68, 72]. Although the mechanism regulating the expression of lactate dehydrogenase subunits and the synthesis of active tetramers is unknown, it is known that the *ldha* and *ldhb* genes are influenced by separate gene control regions [19–23]. The various genetic variants of the A and B subunits [23, 84] observed in humans in no form known to date have been associated with neoplastic transformation, and although there was found an active LDH isoform (LDHK) with different structural and biochemical properties in human sarcomas, it was finally demonstrated that this enzyme, produced by cells infected with Kirsten virus, is fully encoded by the virus and used by it to control transcription [23, 86]. Shim et al. suggest that there are protein products of oncogenes such as myc, ras and src, commonly recognized as factors directly related to the tumor process, that have the ability to activate expression of the *ldhaA* gene [6]. The authors believe that even the LD5 isoform itself, in addition to the known metabolic role, can be functionally involved, in the phosphorylated form, in the modulation of gene expression at the transcriptional level and/or in DNA replication. The fact is that in the case of transformed cells, tyrosine phosphorylation of some proteins, including LDH, which do not function in such forms in normal cells, is observed [87].

Kawamoto found that LDH isozyme levels tended to increase in the order from LD1 to LD5 in both noninvasive and advanced breast cancers compared with normal breast tissues [60]. It was also reported that as the distance from the tumor margin increases, the A/B ratio of LDH subunits decreases and it is associated with decreasing relative activity of LD5 and LD4 isoforms, and increasing LD1 and LD2 [17, 66]. A number of studies have shown that there is a zone around the tumor in which, despite the lack of visible morphological changes, changes in the activity of LDH isoenzymes are observed [19, 20, 66–68]. These authors concluded that the reorientation of LDH isoenzymatic activity in histologically normal tissues surrounding the tumor must be due to an impaired metabolic balance reflecting precancerous changes. This phenomenon, termed the “field effect” (also known as the field defect, field cancerization, or field carcinogenesis), was also observed in a study on the expression of carcinoembryonic antigen (CEA) [88], DNA binding protein (DNA-P) [89] or in the case of analysis of activity of G6PDH (glucose-6-phosphate dehydrogenase), the main enzyme of the pentose cycle [90]. It may suggest that metabolic reprogramming in cancer cells precedes the morphological changes related to malignancy. However, it should be remembered that LDH isoenzyme patterns in tissues are always the resultant of the isoenzymograms of individual components of a given tissue [54].

### Lactate dehydrogenase as a predictive marker

Serum LDH, beyond its diagnostic and prognostic role, has also proved to be a useful indicator of the effectiveness and efficiency of anticancer therapy (Table 1). Many clinical studies have supported the utility of LDH, among others, in the estimation of the likely course and outcome of multiple cancer types. The determination of LDH levels with post-treatment monitoring was strongly recommended by the European Society for Medical Oncology (ESMO) for patients with non-Hodgkin lymphoma (NHL) [75]. Bar et al. found that in patients with advanced colorectal cancers (CRCs), high LDH serum levels strongly correlated with cancer stage and progression-free survival. LDH isoenzyme levels in the serum were tested in patients with colorectal cancers receiving chemotherapy along with the antiangiogenic drugs bevacizumab or cediranib, potent inhibitors of vascular endothelial growth factor receptor (VEGFR) tyrosine kinases. Isoenzymes associated with a hypoxic metabolism turned out to be a negative prognostic marker. Moreover, it was observed that patients with higher expression of hypoxia-related LDH isoenzymes had a trend towards a better outcome from cediranib as compared to bevacizumab. To sum up, the study proved that progression-free survival and overall survival are related to the relative LDH isoenzymes levels, independently of the treatment. In general, it also confirmed that both high levels of hypoxic LDH isoenzymes and high total LDH serum levels can be related to the poor prognosis [69]. Similar conclusions were reported by Anami et al., who found that elevated lactate dehydrogenase level might serve as a highly valuable marker for identifying patients with brain metastases from small cell lung cancer who could have poor survival outcomes. They also observed that higher LDH levels correlated strongly with shorter survival of patients with small-cell lung cancer (SCLC) [73].

Because an elevated LDH level was found to be an unfavorable indicator for survival in cancer patients, it was suggested that lactate dehydrogenase can be used as a marker of tumor aggressiveness. An analysis carried out by Cook et al. revealed that elevated serum LDH levels were associated with a nearly 3-fold increased risk of death in patients with bone metastatic castration-resistant prostate cancer [79]. Furthermore, it was demonstrated that LDH levels highly correlate with survival in patients with bone metastases from breast cancer, and the significance of previously described prognostic factors was corroborated [91].

A study conducted by Inomata et al. showed that an elevated level of plasma LDH is a negative prognostic factor in patients with epidermal growth factor receptor mutation-positive non-small cell lung cancer, being treated with gefitinib or erlotinib. It was also found that patients with higher plasma LDH levels had shorter progression-free survival and overall survival periods in comparison to patients with lower plasma LDH levels [74]. Yu et al. reported corresponding findings, but in the case of pancreatic cancer, and also demonstrated that the LDH levels can guide the treatment for that kind of cancer. The conclusion was that levels of LDH were indeed associated with the systemic inflammatory response and served as a major prognostic predictor of overall survival. Serum LDH levels were shown to predict overall survival in patients with advanced pancreatic cancer after gemcitabine-based palliative chemotherapy, and low serum LDH levels strongly correlated with longer overall survival [70]. Next, it was found that levels of LDH may have significant prognostic value for the response to chemotherapy and survival also in patients with advanced triple-negative breast cancer. The study shows that abnormal baseline LDH levels corresponded to a notably shorter overall survival in comparison to the patients whose LDH baseline levels were normal. The most objective response rate after first-line chemotherapy has been reported in patients whose LDH levels decreased to normal after treatment [62].

Li et al. in 2018 found that variation in serum LDH level could be used as a predictive biomarker of effectiveness of bevacizumab in non-small cell lung cancer (NSCLC) patients [76]. Previously, Sun et al. reported that high levels of L-lactate dehydrogenase B (LDHB) expression in tumor tissue of oral cancer patients treated with paclitaxel could be connected with poor overall survival [82]. On the other hand, L-lactate dehydrogenase A (LDHA) expression levels in tumors have been found to be associated with Ewing’s sarcoma patients’ sensitivity to cetuximab [83]. Moreover, Peliazzari et al. in 2019 confirmed that LDH serum level fluctuations in response to the first-line treatment predicts survival in metastatic breast cancer, while it is well known that the higher the LDH levels are, the shorter is the overall survival rate [63].

On the other hand, it was demonstrated that pretreatment LDH may be used as a potential predictor for immune checkpoint inhibitors (ICIs) in patients with NSCLC and that a high pretreatment LDH level is statistically significantly correlated with poor outcomes of NSCLC patients receiving ICI-based treatment [77]. However, Oya et al. were the first to show that serum CRP and LDH values, as well as performance status, are meaningfully associated with the response duration of nivolumab and survival in advanced NSCLC patients treated with nivolumab. These findings might suggest that serum LDH levels are correlated with the immunotherapy effect. Both immune checkpoint inhibitors and the effectiveness of nivolumab likely depend on both tumor biomarkers and the patient status. Unlike treatment with EGFR-specific and anaplastic lymphoma kinase (ALK)-specific tyrosine kinase inhibitors (TKIs) of advanced NSCLC patients with high levels of CRP, LDH in the serum and poor performance status were found not fit for treatment with nivolumab [78].

Nagamine et al., who focused only on LDHB, found that LDHB might also be used as a marker of cetuximab sensitivity as well as a predictive biomarker for sensitivity to anti-EGFR therapy in colorectal cancers. It was discovered that LDH-B expression was increased in cetuximab-resistant colorectal cancer cell lines, which suggests that LDHB might play a significant role in cancers’ acquisition of drug resistance [71]. Additionally, another interesting discovery was reported by Bilir et al. in 2016; they found a correlation between baseline pain scale scores and serum lactate dehydrogenase levels together with fentanyl dosage. It suggests that serum LDH levels could be used for fentanyl prescription by clinicians for cancer pain in addition to the pain assessment tools [91].

## Conclusions

In conclusion, it is widely recognized that the increased rate of glycolysis in rapidly growing tumor cells is expressed by changes in LDH total activity and/or the LDH isoform composition. Unfortunately, too low specificity and sensitivity of these changes means that LDH cannot be recommended as a specific marker in cancer diagnosis. However, the available data do not exclude the possibility that the measurement of LDH could serve as a valuable auxiliary factor for monitoring the course of certain cancer diseases.

## Data Availability

Not applicable.

## Abbreviations

ALF: Acute liver failure
ATP: Adenosine triphosphate
CEA: Carcinoembryonic antigen
ChoRE: Carbohydrate response element
CRC: Colorectal cancer
CRP: C-reactive protein
DNA-P: DNA binding protein
EGFR: Epidermal growth factor receptor
ELISA: Enzyme-linked immunosorbent assay
Glut1: Glucose transporter-1
G6PDH: Glucose-6-phosphate dehydrogenase
HIF-1/2: Hypoxia inducible factor 1/2
HRE: Hypoxia-responsive element
ICI: Immune checkpoint inhibitor
LDH: Lactate dehydrogenase
LDHi: Lactate dehydrogenase isoenzymes
LD1-LD6 (or LDH1 to LDH5): Lactate dehydrogenase isoenzyme 1 to 6
mRNA: messenger RNA
NAD: Nicotinamide adenine dinucleotide
NHL: Non-Hodgkin lymphoma
NSCLC: Non-small cell lung cancer
real-time PCR: real-time polymerase chain reaction
SCLC: Small-cell lung cancer
TKI: Tyrosine kinase inhibitor
VEGFR: Vascular endothelial growth factor receptor
